# Alcohol Induced Alterations to the Human Fecal VOC Metabolome

**DOI:** 10.1371/journal.pone.0119362

**Published:** 2015-03-09

**Authors:** Robin D. Couch, Allyson Dailey, Fatima Zaidi, Karl Navarro, Christopher B. Forsyth, Ece Mutlu, Phillip A. Engen, Ali Keshavarzian

**Affiliations:** 1 Department of Chemistry and Biochemistry, George Mason University, Manassas, Virginia, United States of America; 2 Department of Medicine, Division of Digestive Diseases and Nutrition, Rush University Medical Center, Chicago, Illinois, United States of America; 3 Department of Biochemistry, Rush University Medical Center, Chicago, Illinois, United States of America; 4 Department of Pharmacology, Rush University Medical Center, Chicago, Illinois, United States of America; 5 Department of Molecular Biophysics and Physiology, Rush University Medical Center, Chicago, Illinois, United States of America; 6 Division of Pharmacology, Utrecht Institute for Pharmaceutical Sciences, Faculty of Science, Utrecht University, Utrecht, The Netherlands; Charité, Campus Benjamin Franklin, GERMANY

## Abstract

Studies have shown that excessive alcohol consumption impacts the intestinal microbiota composition, causing disruption of homeostasis (dysbiosis). However, this observed change is not indicative of the dysbiotic intestinal microbiota function that could result in the production of injurious and toxic products. Thus, knowledge of the effects of alcohol on the intestinal microbiota function and their metabolites is warranted, in order to better understand the role of the intestinal microbiota in alcohol associated organ failure. Here, we report the results of a differential metabolomic analysis comparing volatile organic compounds (VOC) detected in the stool of alcoholics and non-alcoholic healthy controls. We performed the analysis with fecal samples collected after passage as well as with samples collected directly from the sigmoid lumen. Regardless of the approach to fecal collection, we found a stool VOC metabolomic signature in alcoholics that is different from healthy controls. The most notable metabolite alterations in the alcoholic samples include: (1) an elevation in the oxidative stress biomarker tetradecane; (2) a decrease in five fatty alcohols with anti-oxidant property; (3) a decrease in the short chain fatty acids propionate and isobutyrate, important in maintaining intestinal epithelial cell health and barrier integrity; (4) a decrease in alcohol consumption natural suppressant caryophyllene; (5) a decrease in natural product and hepatic steatosis attenuator camphene; and (6) decreased dimethyl disulfide and dimethyl trisulfide, microbial products of decomposition. Our results showed that intestinal microbiota function is altered in alcoholics which might promote alcohol associated pathologies.

## Introduction

Clinical and experimental data have demonstrated that the intestinal microbiota plays a major role in maintaining a healthy state, while an abnormal bacterial community can contribute to the development/progression of various pathological diseases [1]. It is also well established that diet impacts the intestinal microbiota composition and diversity [2]. Alcohol is a major component of diet in Western societies, which could potentially impact the intestinal microbiota community. Several studies, including our own, have shown that excessive alcohol consumption impacts the intestinal microbiota composition in both rodent models and humans, causing disruption of intestinal microbiota homeostasis (dysbiosis) [3–6]. The changes in the intestinal microbiota community may be a potential co-factor for the development of tissue injury and organ pathologies associated with excessive alcohol consumption, such as alcoholic steatohepatitis and cirrhosis (alcoholic liver disease (ALD)). Several epidemiologic and observational studies show that only a subset of alcoholics develop organ damage such as ALD, indicating that while chronic alcohol consumption is necessary, it is not sufficient to cause organ dysfunction [7,8]. Additional experimental studies indicate that proinflammatory, gut derived bacterial products like endotoxins (lipopolysaccharide; LPS) are required co-factors for alcohol-induced organ pathologies like ALD [9–11]. Further, human and experimental studies show that gut leakiness to LPS is one of the primary mechanisms of endotoxemia [12] and abnormal intestinal bacterial community composition (dysbiosis) that has been shown to occur in the subset of alcoholics and alcohol fed rodents [3,5] that can play a major role in oxidative stress, gut leakiness and endotoxemia and thus could potentially cause the development of alcohol-induced pathologies like ALD [12–17].

However, the observed change in the microbiota composition in alcoholics is not indicative of the dysbiotic intestinal microbiota function that could result in the production of injurious and toxic products. Thus, knowledge of the effects of alcohol on the intestinal microbiota function and their metabolites is warranted to complement the results of alcohol-induced changes to the intestinal microbiota composition, in order to better understand the role of the intestinal microbiota in alcohol associated organ pathologies. This knowledge is essential for identifying the potential intestinal microbiota directed therapeutic target(s) to prevent and treat alcoholic organ damage like ALD. However, to the best of our knowledge, there has not been a comprehensive report of the impact of alcohol consumption on the intestinal microbial metabolites.

Recent advancements in the field of metabolomics provide the opportunity to interrogate the impact of alcohol consumption on bacterial metabolites such as volatile organic compounds (VOC) in the stool of alcoholics. Related by their volatility at ambient temperatures, the VOCs comprise a large and structurally diverse family of carbon-based molecules, of both natural and man-made origin. Specialized sampling methods, such as headspace solid-phase microextraction (hSPME), greatly enable the isolation of VOCs from a wide array of biological samples [18–21], including feces [22–27]. hSPME typically involves the partitioning of the VOCs from the headspace above a sample into a polymeric sorbent adhered to a fused silica rod (fiber), subsequent desorption of the VOCs into the heated inlet of a gas chromatograph, separation of the VOC mixture by gas-liquid partition chromatography, and detection by mass spectrometry. Spectral comparison to a reference database enables VOC identification.

One of the challenges to interrogating microbiota metabolites is the selection of the appropriate samples and the method of sample collection in order to avoid potential confounding factors, such as the continual bacterial metabolic events *ex-vivo* after samples, like stool, are voided and exposed to ambient environment before freezing. Indeed, we recently reported that the VOC metabolome derived from stool collected at home was different than that obtained from stool collected during endoscopy and immediately frozen avoiding any *ex-vivo* metabolic events [28].

Here, we report the results of a differential metabolomic analysis comparing VOC metabolomes derived from the stool of alcoholics and non-alcoholic healthy controls. We performed the analysis with fecal samples collected after passage (patient’s home) and then frozen after a period of time, as well as with fecal samples collected directly from the sigmoid lumen (via un-prep sigmoidoscopy) then immediately frozen to prevent metabolic events from occurring after stool collection. Regardless of the approach to fecal collection, we found a stool VOC metabolomic signature in alcoholics that is different from healthy controls.

## Materials and Methods

### Fecal samples

The Institutional Review Boards at George Mason University and Rush University Medical Center approved this investigation. An informed written research consent was signed by all study participants. Fecal samples were endoscopically collected from 18 healthy and 16 alcoholic subjects (the ‘endoscopy collected samples’) or were collected *ex vivo* after passage from 25 healthy and 22 alcoholic subjects (the ‘home collected samples’), in the manner described below. (Table 1) depicts the demographic characteristics of the study subjects. Each subject completed a detailed health questionnaire that showed that healthy participants did not have any chronic GI or systemic disease or symptoms, none were taking any regular medication except for blood pressure and cholesterol, and none used supplements including probiotics or prebiotics. No subject took antibiotics, for at least three months, and none of the healthy participants were excessive drinkers of alcohol (women consumed less than 2 drinks per sitting per day or no more than 7 drinks per week and men consumed no more than 4 drinks per sitting per day or no more than 14 drinks per week.). Women were considered alcoholics if they consumed 4 or more drinks per day or 8 or more drinks per week, while men were considered alcoholics if they consumed 5 or more drinks per day or 15 or more drinks per week. All study participants were instructed not to change their usual dietary consumption and, as verified by a dietary questionnaire, all participants demonstrated no change in their typical diet or health status during and 7 days prior to stool collection. We compared the dietary consumption of the healthy and alcoholic cohorts and found no substantial differences between cohorts.

**Table 1:** Characteristics of the study participants.

	Alcoholics	Healthy Controls	Alcoholics	Healthy Controls
	Endo Collection	Endo Collection	Home Collection	Home Collection
	(N = 16)	(N = 18)	(N = 22)	(N = 25)
**Gender:** **male, M; female, F**	15 M; 1 F	8 M; 10 F	18 M; 4 F	11 M; 14 F
**Race:** **Caucasian, C; African American, AA;Asian, A**	8 C; 8 AA	9 C; 8 AA; 1 A	10 C; 12 AA	12 C; 12 AA; 1 A
**Age Range**	30–64	20–63	30–64	20–63
**Age Mean**	49.9	39	48.4	37.7
**BMI Range**	15.9–43.9	19.6–45.4	15.9–43.9	19.6–45.4
**BMI Mean**	25.3	31.6	27.6	29.7
**Alcohol Consumption History (Years) Mean**	28.9	12.9	27.4	12
**Smoking During Time of Study (1–2 packs per day)**	8 out of 16	5 out of 18	11 out of 22	6 out of 25
**NSAID Usage During Time of Study (Daily)**	3 out of 16	0 out of 18	4 out of 22	0 out of 25

Study participants in the endoscopy collected group had their stool collected *in vivo* via un-sedated sigmoidoscopy, after providing an informed, written consent. There was no colon preparation prior to sigmoidoscopy. The stool in the lumen of the distal sigmoid was obtained using a Roth Net (US Endoscopy, Mentor, OH), removed with the sigmoidoscope, and then placed in a cryovial and immediately snap frozen in liquid nitrogen. Upon removal from the liquid nitrogen, the cryovial was immediately stored in a -80°C freezer until analysis. For the home collected group, study participants were instructed on how to place their stool into a BD Gaspak EZ Anaerobe Gas Generating Pouch System with Indicator (Becton, Dickinson and Company, Sparks, MD), to minimize the exposure of stool to high oxygen ambient atmosphere. Study subjects were asked to keep the sealed anaerobic stool bag in a cold environment until bringing the anaerobic stool bag to the hospital. Upon receipt, the stool was immediately stored in a -80°C freezer. The interval between passage of stool and storage at -80°C was within 12 to 24 hours.

### hSPME procedure

The frozen fecal samples were dispensed in 0.2 g aliquots into 4 mL WISP style screw thread amber glass vials, sealed with Black Top Hat PTFE/Silicone caps (J.G. Finneran, Vineland, NJ), and stored at -80°C until analyzed. Three different SPME fibers (Supelco, Bellefonte, PA) were used in our investigation; 75 μm carboxen-polydimethylsiloxane (CAR-PDMS), 85 μm polyacrylate (PA), and 50/30 μm divinylbenzene (DVB)-CAR-PDMS. Each study subject’s fecal sample was extracted with each of the three SPME fibers, using a new fecal aliquot for each hSPME. All fibers were preconditioned before use, following the manufacturer’s instructions. All analyses were performed in duplicate. The sample vials were heated to 60°C for 30 minutes prior to positioning the hSPME fiber into the headspace above the feces. The extraction was performed until equilibrium (18 hours; [26,28]), with the sample vial temperature held at 60°C for the duration of the extraction. The fiber assembly was then placed into the GC inlet for thermal desorption of the analytes.

### GC-MS Instrument

Samples were analyzed using an Agilent 7890A GC equipped with a DB5-MS capillary column (Agilent, Palo Alta, CA; 30 m length, 0.25 mm ID, and 0.25 μm film thickness), a 0.75 mm ID SPME injection port liner, and a 5975 inert XL mass selective detector (MSD) with triple axis detector. The GC injection port was operated in splitless mode at select inlet temperatures, dependent upon the SPME fiber used (300°C, CAR-PDMS; 280°C PA; 270°C DVB-CAR-PDMS). Helium carrier gas was set to a flow rate of 1.17 mL/min. The GC oven was held at an initial temperature of 35°C for 1 min, ramped at 3°C/min to 80°C, then 10°C/min to 120°C, and finally 40°C/min to 260°C, where the temperature was held for 1.5 min. The total run time for the analysis was 25.0 min. The MSD was scanned from 30 to 550 amu at a rate of 2.81 scans/sec.

### Data processing and analysis

The VOCs were identified in the GC-MS chromatograms using the National Institute of Standards and Technology (NIST, Washington, DC) Automated Mass Spectral Deconvolution and Identification System (AMDIS, ver. 2.69) software and mass spectral library (NIST08). Compounds with 85% or greater probability of match to a molecule in the NIST08 library were only considered. Each AMDIS outfile, containing a list of identified metabolites and their corresponding peak height values, was filtered using custom Perl scripts designed to remove background analytes (e.g. siloxanes) and eliminate metabolite redundancies (retaining the replicate with the highest peak value). Duplicate sample data sets were combined using Perl scripts created to merge AMDIS outfiles and average the corresponding peak height values. A comprehensive, three-fiber metabolite dataset was prepared for each sample by pooling the metabolites obtained using the CAR-PDMS, PA, and DVB-CAR-PDMS fibers and summing the corresponding peak height values (a peak height of zero was imputed for missing metabolites). A Perl script was then used to assemble two complete metabolite matrices; one containing all of the endoscopy collected healthy and alcoholic patient samples and their accompanying metabolites, and another containing all of the home collected healthy and alcoholic patient samples and their accompanying metabolites. Metabolites present in ≤20% of the samples were treated as one-offs and were removed [28]. Each metabolite matrix was arranged into two cohorts (healthy and alcoholic) and the outlier peak height values were identified in each cohort using a plot of (mean-median)/median for each analyte and a cutoff value ≥1.5. Outliers were replaced with the median value for that metabolite within the cohort. Metabolite peak height values were then standardized across the two cohorts by conversion to Z-scores (peak height-mean/standard deviation). A Pearson (n) principal component analysis was then performed using the standardized metabolite matrices and the statistical package XLSTAT 2012.6.02. XLSTAT was also used to perform two sample T tests between cohorts for each metabolite. Benjamini-Hochberg critical values were calculated as (i/m)Q, where i is the rank in an ascending list of *p* values, m is the total number of tests, and Q is a false discovery rate of 0.15. Pearson’s correlation coefficients were calculated using Microsoft Excel. A correlation network was created using the R statistical package. Unsupervised hierarchical clustering and heatmap generation was accomplished using R, with the Manhattan method and Pearson correlation for the distance measure. Fold change calculations were performed using Microsoft Excel. Custom Perl scripts were used to combine and compare the cohort metabolites to identify the common and unique metabolites and to group the metabolites and their relative abundance into defined chemical classes. Bar graphs and ROC curves were prepared using GraphPad Prism ver. 4.0.

## Results and Discussion

To determine if the fecal VOC metabolome composition is altered by excessive alcohol consumption, we obtained a combined total of 81 stool samples from healthy and alcoholic volunteers. As we illustrated previously [28], the approach to collecting a fecal sample has an impact on the derived VOC metabolome, so we elected to acquire the fecal samples in each of two ways; *in vivo* by endoscopy and *ex vivo* by home collection after passage, as detailed in Materials and Methods. The VOCs from the collected samples were extracted by hSPME and identified by GC-MS. To ensure greater metabolome coverage while still accommodating reasonable sample throughput, three different hSPME fiber chemistries were used (CAR-PDMS, PA, and DVB-CAR-PDMS). All of the extractions were performed in duplicate (using different fecal aliquots) and the replicates combined by averaging the chromatographic peak height values. Hence, a total of 486 chromatograms were generated from the 81 participant fecal samples, resulting in both endoscopy collected (containing 16 alcoholic samples and 18 healthy samples) and home collected (containing 22 alcoholic and 25 healthy samples) VOC metabolome datasets.

**Fig 1:**
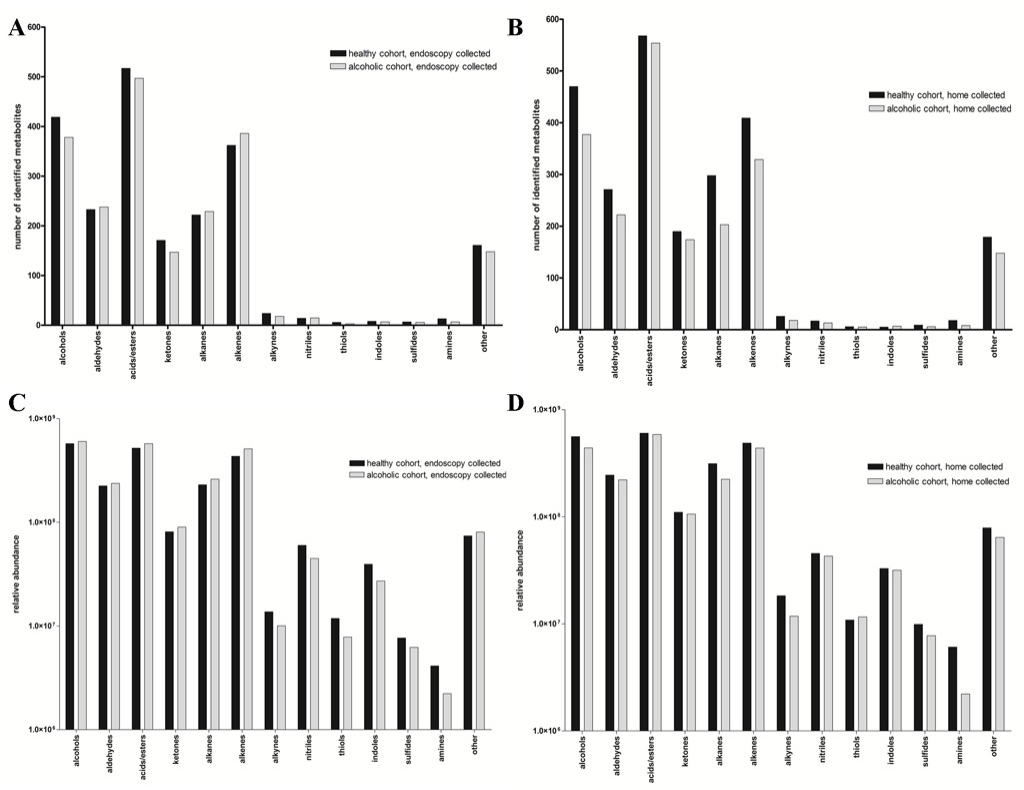
Metabolite composition and abundance. The pooled analytes present in the alcoholic and healthy cohorts were distributed among the listed chemical classes and then tallied. A and B) The bar graphs indicate the total number of analytes in each chemical class for the endoscopy (A) or home collected (B) fecal VOC metabolomes. C and D) The relative abundance (peak height) of the metabolites present in each cohort were distributed among the indicated chemical classes and then summed. The bar graphs indicate the relative abundance of each class for the endoscopy (C) or the home collected (D) fecal VOC metabolomes.

When constraining metabolite identification to a minimum 85% molecular library match, a grand total of 2,659 different VOCs are identified in the endoscopy collected fecal samples. In contrast, the home collected samples collectively contain 2,883 total analytes, an additional 224 analytes relative to the endoscopy group. Fig. 1 presents a comparison of the alcoholic and healthy cohort composition in terms of the number of identified analytes and the relative abundance in each of the indicated chemical classes. Of greatest significance, in both the endoscopy collected and home collected VOC metabolomes, the overall chemical distribution appears similar among the healthy and alcoholic cohorts (Figs. 1A and 1B), with a slight bias towards alcohols, alkanes, and alkenes in the home collected healthy group. Further, regardless of the means by which the feces was isolated, very little difference in each of the chemical classes is apparent when comparing relative analyte abundance between the healthy and alcoholic cohorts (Figs. 1C and 1D). Additionally, while the metabolome composition as a whole is asymmetrically distributed across the various chemical classes, the relative distribution remains consistent regardless of the cohort or means by which the feces were collected (e.g. the acids/esters group always has the greatest number of metabolites, followed by the alcohols and alkanes, and so on). While this latter observation may simply be a reflection of the three fiber hSPME technique (fiber chemistry dictates the nature of the isolated analytes and while a three fiber analysis expedites sample processing, it results in an incomplete metabolome relative to a study using five or more different fibers [26]), this distribution is also suggestive of a global homeostatic relationship among the chemical classes within the feces. Additional work is required to further explore this possibility.

**Fig 2:**
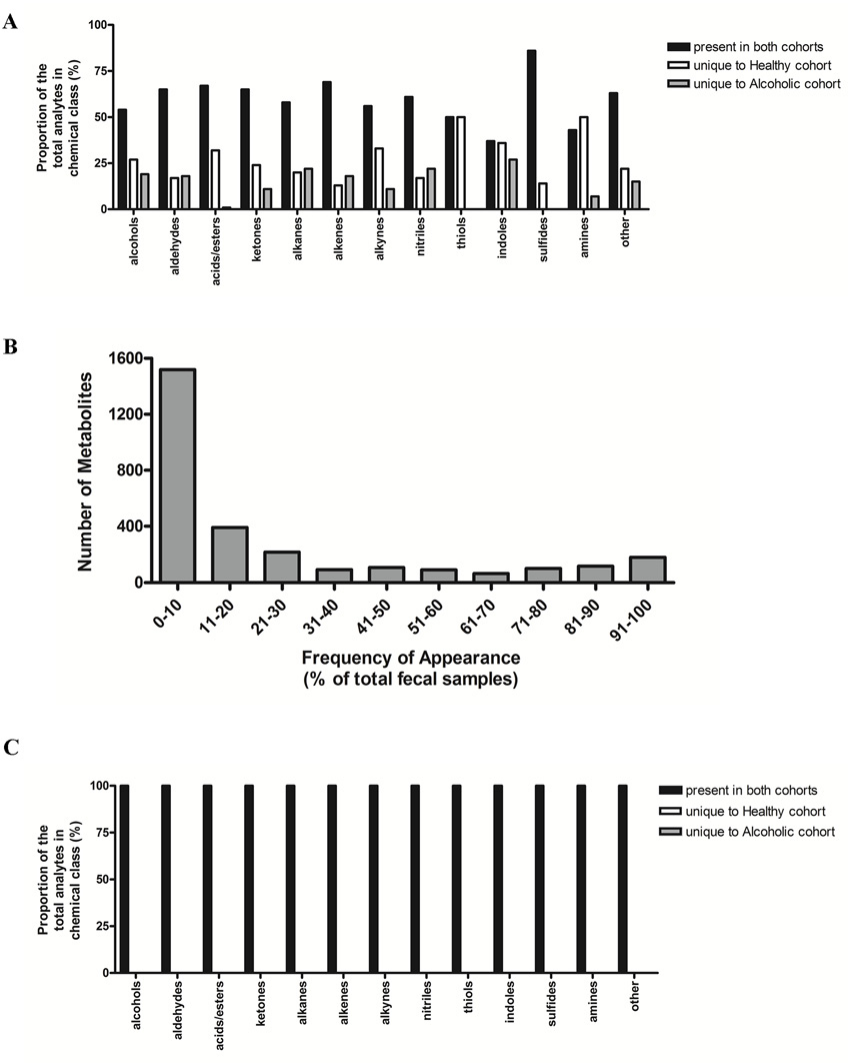
Metabolite distribution within the endoscopy collected fecal samples. A) The VOCs identified in the fecal samples were sorted according to the indicated chemical classes and then further arranged by their unique association with either the healthy or alcoholic cohorts, or their appearance in both cohorts. The percent distribution relates to the total number of metabolites within the chemical class. B) The number of identified VOCs as a function of frequency of appearance among the total number of fecal samples analyzed. A large number of analytes appear in a small number of fecal samples, likely a reflection of dietary variation among the study participants. C) The plot was prepared as described in A), but with the exclusion of the low frequency metabolites (≤20%) identified in B). Consequently, there are no longer any metabolites exclusive to either the healthy or alcoholic cohorts.

Although Fig. 1 suggests that the overall chemical composition is very similar between the healthy and alcoholic cohorts, noteworthy differences become apparent when performing a higher resolution comparison of the specific analytes identified within each of the chemical classes. Fig. 2A presents the similarities and differences within the endoscopy collected VOC metabolomes. While a significant number of metabolites are common to both of the cohorts, in most of the chemical classes a substantial number are uniquely associated with either the healthy or alcoholic samples (equivalent results are also obtained when comparing the home collected metabolomes (data not shown)). However, all of these unique analytes appear in only a small proportion (20% or fewer) of the total number of stool samples analyzed (Fig. 2B). Hence, these ‘cohort-unique’ metabolites are most likely attributed to variations in dietary intake [28], and when these low frequency metabolites are excluded, the combined metabolome composition appears identical among the cohorts (Fig. 2C). Alternatively, since only a subset of alcoholics develop organ damage such as ALD [7,8], it is also possible that these low frequency metabolites comprise a unique VOC signature associated with eventual organ dysfunction. However, since our investigation is cross sectional by design, we cannot determine if the subset of alcoholics with the unique VOC metabolites will go on to develop organ damage. An additional longitudinal study is required to address this possibility.

**Fig 3:**
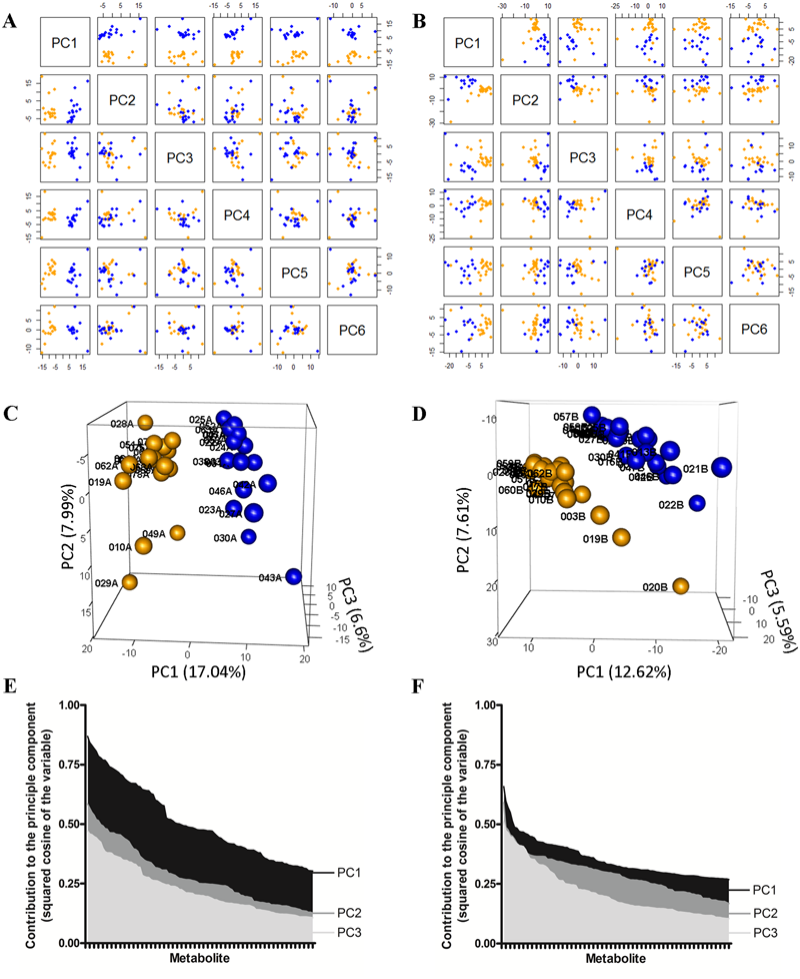
Principal component analysis (PCA) of the VOC metabolomes derived from the healthy and alcoholic human fecal samples. Infrequent metabolites were disregarded by restricting the analysis to analytes that appear in a minimum of 21% of all samples in each cohort (see Fig. 2). Hence, the endoscopy collected dataset contains 525 metabolites while the home collected dataset contains 641. A) PCA plots of the endoscopy collected fecal VOC metabolome. The first (PC1) through sixth (PC6) principal components are shown, relative to one another. Healthy samples are identified as blue spheres, while alcoholic samples are denoted as yellow spheres. The two cohorts clearly segregate in all plots involving PC1, while little to no difference is observed among the cohorts in the combinatorial plots with PC2 through PC6. B) PCA plots of the home collected fecal VOC metabolomes. The samples are colored as described in A). The two cohorts segregate in all plots involving PC1, PC2, and PC3 (to differing degrees), while segregation is not observed in the combinatorial plots of PC4, PC5, and PC6. C) A three dimensional PCA plot of the endoscopy collected fecal VOC metabolome (depicting the first, second and third principal components) clearly illustrates the distinctiveness of the healthy and alcoholic cohorts. Samples are colored as in A). Unique sample identifiers are shown adjacent to each data point. D) A three dimensional PCA plot of the home collected fecal VOC metabolome clearly differentiates the healthy and alcoholic cohorts. E and F) Metabolite contribution to the principal components. For clarity, each graph is restricted to the first three principal components and the top 104 contributing metabolites. Metabolites were arranged by descending contributions to each principal component, and the values plotted in the bar graph. The plots indicate that numerous metabolites collectively contribute to cohort segregation in the endoscopy collected (E) and home collected (F) fecal VOC metabolomes. See text for further discussion.

**Fig 4:**
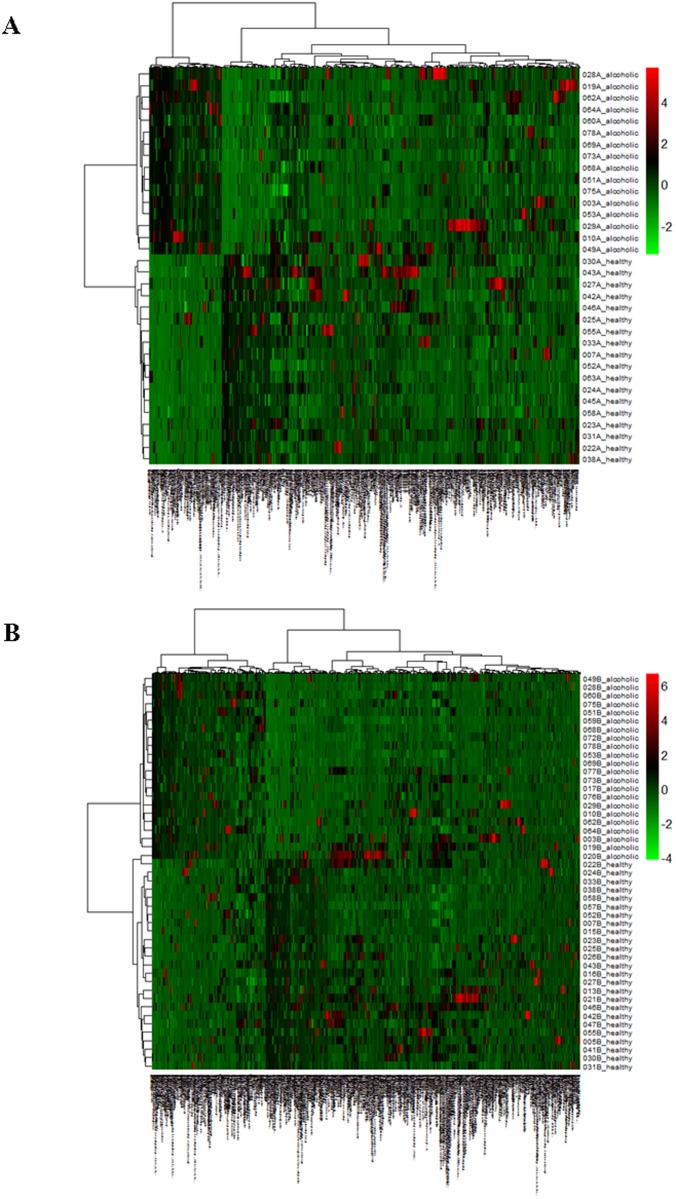
Heat map showing the unsupervised hierarchical clustering of the fecal samples according to the similarity of metabolome composition. The endoscopy collected fecal metabolomes are compared in (A) while the home collected fecal metabolomes are compared in (B). The samples are arranged in rows, the metabolites in columns, and shades of red represent elevation of a metabolite while shades of green represent decrease of a metabolite, relative to the median metabolite levels (see color scale). In the dendrograms, the clustering clearly differentiates the alcoholic and healthy fecal samples.

**Fig 5:**
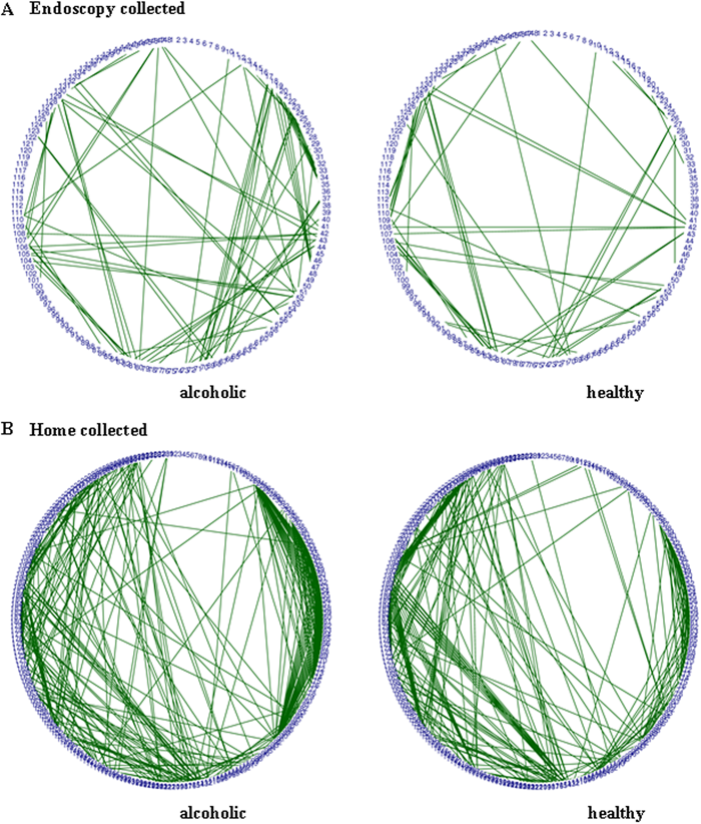
Metabolite correlation network of the endoscopy collected (A) and home collected (B) fecal VOC metabolomes. Pearson’s correlation coefficients were calculated for metabolites present in 80% or greater of the total fecal samples. A Pearson correlation value greater than 0.95 is depicted as a green line between metabolites (negative correlations are not shown, as correlation values less than-0.95 were not obtained). To facilitate comparison of the networks, metabolites are numerically represented and their placement around the circumference of each network is fixed among the paired plots. Regardless of the fecal collection method used, the fecal samples from the alcoholic participants have a notably different correlation network than that seen in the fecal samples from non-alcoholics. This difference is even more apparent in correlation networks derived using metabolites present in ≥21% of all fecal samples (S2 Fig.).

**Fig 6:**
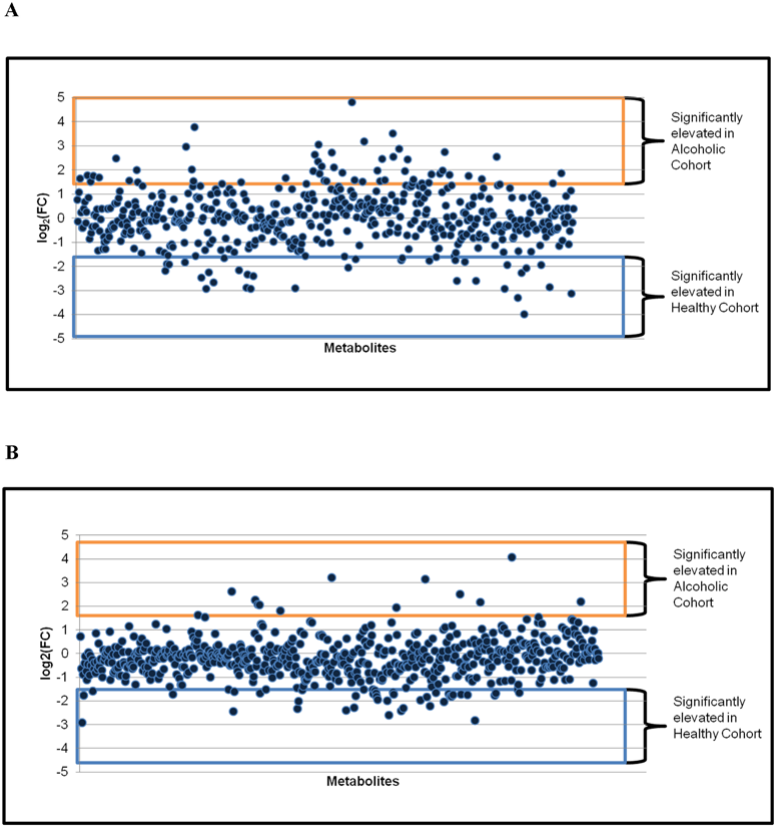
Fold change analysis of the metabolite abundance between the healthy and alcoholic fecal samples. The fold change (FC) is calculated as the log transformation of the ratio between the mean metabolite abundance in the alcoholic cohort relative to the healthy cohort. The analysis was performed with both the endoscopy (A) and home passage collected (B) metabolomes. A log_2_(FC) greater than 1.5 is deemed significant (equivalent to a threefold or greater change in metabolite abundance). A fold change analysis comparing median analyte values produces similar results (not shown).

We have indicated previously how the colonic microbiome is altered in alcoholism [5]. To ascertain how the metabolite composition and abundance relates among the healthy and alcoholic cohorts, a principal component analysis (PCA) was performed (restricted to analytes appearing in >20% of the samples). As seen in Figs. 3A through 3D, the PCA clearly segregates the healthy and alcoholic samples based upon their VOC metabolome composition, regardless of the approach to fecal sample acquisition. With the endoscopy collected samples (Figs. 3A and 3C), the first principal component clearly discriminates between the two cohorts (as evidenced by the samples segregating into separate groups along the PC1 axis of the PCA plots), whereas the second and third components reveal variation within each of the two segregated cohorts (particularly evident with the alcoholic samples 010A, 029A, 049A and healthy samples 023A, 027A, 030A, 042A, 043A, and 046A (Fig. 3C)). Cohort differentiation is also apparent in the home collected fecal VOC dataset, with healthy and alcoholic segregation readily apparent along the PC1, PC2, and PC3 axis (Figs. 3B and 3D). Numerous metabolites collectively contribute to the segregation of the healthy and alcoholic cohorts (as ranked by the squared cosine of the variable, Figs. 3E and 3F), the top ten of which alone cause segregation of the healthy and alcoholic samples in a PCA (S1 Fig.). A dendrogram and accompanying heat map further depict the clear differentiation of the healthy and alcoholic fecal VOC metabolomes (Fig. 4). Additionally, metabolite correlation networks derived from the healthy and alcoholic fecal VOC metabolomes also illustrate extensive alcohol related changes to the relationships among the metabolites (Fig. 5 and S2 Fig.). Further, a fold change analysis of the endoscopy and home collected fecal VOC metabolomes highlights several metabolites that undergo a significant abundance change associated with the excessive consumption of alcohol (Fig. 6).

**Fig 7:**
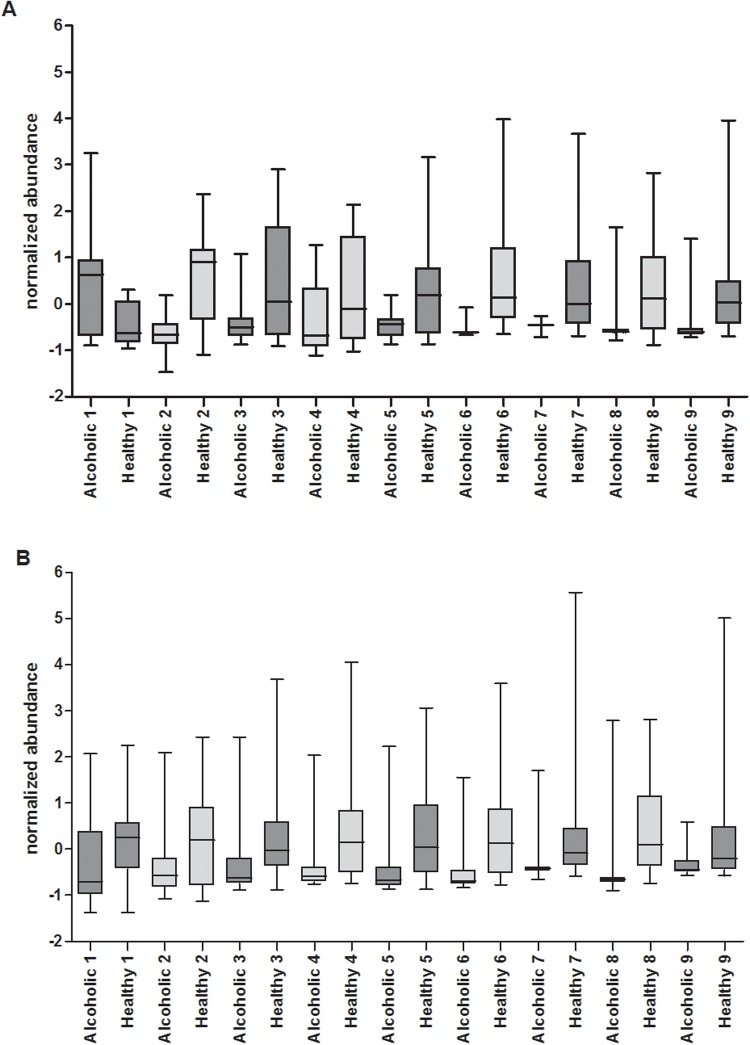
Metabolites with a statistically significant difference in median abundance levels. The metabolites listed in Table 2 were compared among the healthy and alcoholic cohorts. Box plots are shown, depicting the interquartile range of Z-score normalized abundance values, with whiskers extending from minimum to maximum. The median value is identified by a horizontal line within the box. Metabolites from the endoscopy collected samples are shown in A), and are numerically coded as follows; 1) tetradecane, 2) 2-tetradecen-1-ol, 3) 1-undecanol, 4) propanoic acid, 5) cyclopropane, nonyl-, 6) 6-pentadecen-1-ol, 7) 8-Tetradecen-1-yl acetate, 8) 1,15-Pentadecanediol, and 9) Eicosen-1-ol. Metabolites from the home collected samples are shown in B), and are numerically coded as follows; 1) caryophyllene, 2) 1-naphthalenol, 3) phellandrene, 4) dimethyl disulfide, 5) dimethyl trisulfide, 6) camphene, 7) 2,5-pyrrolidinedione, 1-(benzoyloxy)-, 8) 5-hepten-2-one, 6-methyl-, and 9) (2-aziridinylethyl)amine. See text for further discussion.

As listed in Table 2, when restricting the comparison to only those metabolites found in ≥80% of the total samples present in either the alcoholic or healthy fecal cohort, of a total of 152 metabolites, 18 demonstrate a statistically significant difference in abundance between the healthy and alcoholic cohorts (*p* value <0.05 and fold change greater than 2), 9 of which are associated with the endoscopy collected samples and 9 are associated with the home collected samples. A PCA with these analytes alone clearly differentiates the healthy and alcoholic cohorts from one another, with either of the fecal collection techniques (not shown). Fig. 7 presents box plots comparing the abundance of these metabolites in the alcoholic and healthy control fecal samples. Of significance, tetradecane demonstrates a fourfold increase in its median abundance level in the endoscopy collected alcoholic fecal VOC metabolome, relative to the healthy controls (see Fig. 7A and Table 2). On the other hand, tetradecane has only a 1.1 fold increase in median abundance in the home collected alcoholic fecal VOC metabolome (data not shown). This discrepancy between the endoscopy and home collected samples corroborates our previous report that the approach to fecal sample acquisition can have a significant impact on the resulting derived VOC metabolome [28]. As the endoscopy collected samples are immediately snap frozen in liquid nitrogen after their *in vivo* isolation, these samples best reflect the *in situ* fecal VOC metabolome and avoid possible *ex vivo* fermentation/evaporation effects that may influence/alter the composition of the home collected fecal samples. Hence, the endoscopy collected samples are preferred. However, in light of the significantly increased cost associated with the endoscopy collection, the relative ease by which the home collected samples are obtained, and the fact that the home collected healthy and alcoholic cohorts are clearly segregated by principal component analysis of their corresponding VOC metabolomes (Fig. 3), we elected to continue our examination of the home collected samples, as they still offer insight into alcohol induced changes to the fecal VOC metabolome, and implicate biomarkers indicative of alcoholism.

**Table 2:** Metabolites with a statistically significant difference in abundance between the healthy and alcoholic cohorts (*p*<0.05; *p*<the Benjamini-Hochberg critical value at a false discovery rate of 0.15).

Endoscopy Collected Fecal Samples					
increased in alcoholics	p value	fold change	frequency in Healthy (of 18)	frequency in Alcoholic (of 16)	area under ROC curve
Tetradecane	0.013	4.07	15	13	0.68
**decreased in alcoholics**					
2-Tetradecen-1-ol	0.000	2.52	17	14	0.83
1-Undecanol	0.025	2.14	15	12	0.67
Propanoic acid	0.029	2.32	18	16	0.67
Cyclopropane, nonyl-	0.012	2.34	14	12	0.70
6-Pentadecen-1-ol	0.002	8.48	14	11	0.85
8-Tetradecen-1-yl acetate	0.008	2.33	14	7	0.77
1,15-Pentadecanediol	0.016	3.09	16	9	0.72
Eicosen-1-ol	0.035	3.98	14	10	0.74

**Home Collected Fecal Samples**					
increased in alcoholics	p value	fold change	frequency in Healthy (of 25)	frequency in Alcoholic (of 22)	area under ROC curve
none					
**decreased in alcoholics**					
caryophyllene	0.044	2.12	24	20	0.68
1-Naphthalenol	0.023	2.12	25	19	0.64
Phellandrene	0.022	3.12	21	17	0.70
Dimethyl disulfide	0.016	4.63	24	22	0.80
Dimethyl trisulfide	0.002	4.27	25	22	0.77
Camphene	0.012	5.56	20	16	0.80
2,5-Pyrrolidinedione, 1-(benzoyloxy)-	0.040	2.23	20	12	0.71
5-Hepten-2-one, 6-methyl-	0.033	3.86	21	14	0.75
(2-Aziridinylethyl)amine	0.030	2.55	21	16	0.66

As mentioned, tetradecane is increased in abundance in the endoscopy collected alcoholic fecal samples, relative to the healthy controls. Additionally, the major contribution of tetradecane to cohort segregation is within the first principal component of the PCA plot (Fig. 3C), as indicated by the squared cosine of the variable (Fig. 3E). A member of the acyclic alkane family of molecules, tetradecane has been identified as a breath biomarker of oxidative stress [29]. Hence, in the context of fecal VOCs, the increased abundance of tetradecane in the alcoholic cohort might be indicative of a state of oxidative stress within the gut. Accordingly, alcohol-induced oxidative stress is associated with the development of gut hyperpermeability, endotoxemia, and subsequent alcoholic steatohepatitis [30].

On the contrary, the observed change in relative abundance of tetradecane in the home collected alcoholic cohort samples is only minimal (with a 1.1 fold increase in alcoholics). In fact, no metabolites appearing in ≥80% of the home collected samples demonstrate a 2 fold or larger increase in abundance in the alcoholic samples (rather, the metabolites in the home collected samples demonstrating greater than 2 fold change in abundance were all decreased in alcoholics, as described below). As a point of interest, ascorbic acid 2,6-dihexadecanoate, a known phytochemical [31–33], was detected in 20 of the 25 healthy and 18 of the 22 alcoholic home collected fecal samples and demonstrates a 1.72 fold increase in the alcoholic cohort (*p* value = 0.02; data not shown). Ascorbic acid 2,6-dihexadecanoate is a known constituent of wine [34].

Of the eight metabolites identified as decreased in the endoscopy collected alcoholic fecal samples (Table 2), five are members of the fatty alcohol family of molecules (2-Tetradecen-1-ol, 1-undecanol, 6-pentadecen-1-ol, 1,15-pentadecanediol, and eicosen-1-ol). The first four of these fatty alcohols contribute maximally to segregation of the healthy and alcoholic cohorts along principal component 1 in the PCA (Fig. 3C), while eicosen-1-ol maximally contributes to principal component 3. As depicted in Fig. 7, of these five fatty alcohols, the abundance of 6-pentadecen-1-ol, 1,15-pentadecanediol, and eicosen-1-ol are dramatically suppressed in the alcoholic fecal samples. In fact, these three metabolites are below their detection limits in several of the alcoholic fecal samples (ranging from five samples for 6-pentadecen-1-ol to seven for 1,15-pentadecanediol; see Table 2). *In vivo*, fatty alcohols are obtained from the diet or may be derived from fatty acids and fatty aldehydes via the fatty alcohol cycle [35]. Fatty alcohols are precursors for the biosynthesis of wax esters and ether glycerolipids such as the plasmalogens [35,36]. Of particular relevance, the plasmalogens play an important role in cell-cell interactions and gap junctions, and are known to protect against reactive oxygen species [37]. Further, rodents fed a diet rich in fatty alcohols show elevated plasmalogens in the liver [38]. Hence, it is interesting to speculate that the decreased abundance of these fatty alcohols in the alcoholic cohort might be a response to the alcohol-induced oxidative stress, causing an increase in the biosynthesis of plasmalogens (thereby a decrease in these particular fecal fatty alcohols). On the other hand, low levels of these fatty alcohols in alcoholics might represent low capacity of alcoholics to synthesize plasmalogens and thus render the intestine and liver more susceptible to alcohol-induced oxidative stress. While these fatty alcohols appear to be potential biomarkers of excessive alcohol consumption (particularly 6-pentadecen-1-ol), further investigation is needed to elucidate the details underlying the relationship between alcohol consumption, fecal fatty alcohol abundance, and the plasmalogens.

The level of 8-tetradecen-1-yl acetate in the endoscopy collected samples also appears to be significantly depleted due to excessive alcohol consumption (*p* value = 0.008; Table 2), although the biological meaning remains unclear. Common to most of the healthy samples, its median abundance is decreased 2.33 fold in the alcoholic fecal cohort, even falling below detection limits in over half of the cohort samples (Table 2 and Fig. 7). Nonylcyclopropane, a known phytochemical [39] and VOC associated with meat [40], is also significantly suppressed in the endoscopy collected alcoholic fecal cohort (*p* value = 0.012; 2.34 fold decrease in abundance in the alcoholic cohort relative to healthy). However, as with 8-tetradecen-1-yl acetate, the biological relevance of this decline in abundance is not readily apparent and requires further investigation.

**Fig 8:**
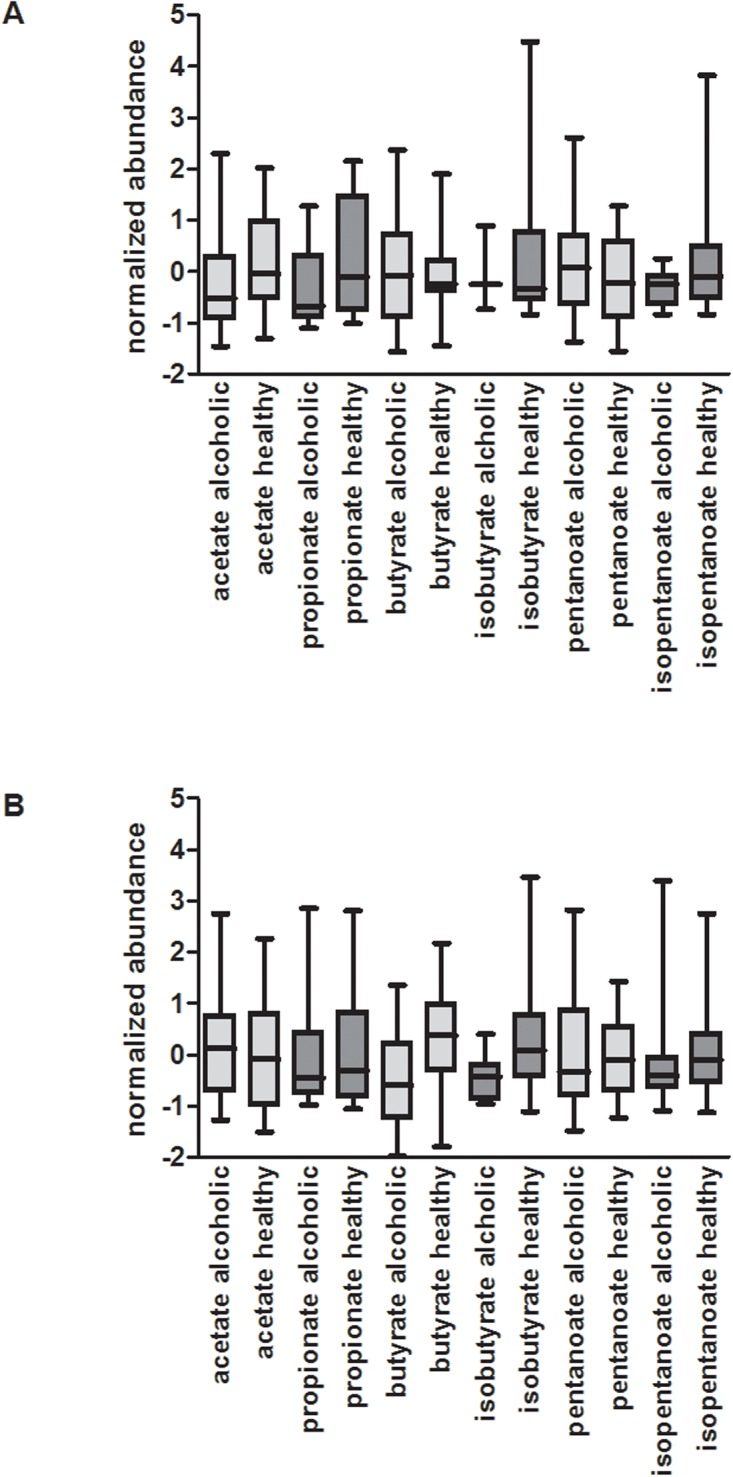
Comparison of SCFA abundance among the cohorts. Box plots are presented, depicted as described in Fig.8. A) Among the endoscopy collected samples, while most of the SCFAs are comparable in abundance within the healthy and alcoholic cohorts, propanoic acid demonstrates a statistically significant reduction in median abundance in the alcoholic cohort relative to the healthy cohort (*p* = 0.03, fold change = 2.32). B) In the home collected samples, butyrate has a 1.6 fold reduction in median abundance, while the remaining SCFAs are very similar between the healthy and alcoholic fecal samples. See text for further discussion.

As indicated in Table 2 and Fig. 7A, the median abundance of propanoic acid is significantly lower in the endoscopy collected alcoholic cohort, relative to the healthy controls (*p* value = 0.029; fold change = 2.32). Short chain fatty acids (SCFAs) such as acetate, propionate, butyrate, isobutyrate, pentanoate, and isopentanoate are products of microbial fermentation and are often considered to be essential to maintain and promote normal colonic epithelial cell barrier integrity [41,42]. Fig. 8 illustrates box plots depicting the abundance of these SCFAs, in both the healthy and alcoholic cohorts. With the endoscopy collected fecal samples (Fig. 8A), among all these SCFAs, only propanoic acid shows a statistically significant reduction in median abundance in the alcoholic cohort relative to the healthy cohort (acetate follows with a 1.49 fold reduction in median abundance and a *p* value of 0.34). In contrast, propanoic acid levels in the home collected alcoholic fecal samples are only reduced by 1.2 fold (*p* = 0.40; Fig. 8B). While the median abundance of isobutyrate appears comparable in the endoscopically collected healthy and alcoholic cohorts, it’s notable that this SCFA was only detected in 8 of the 16 alcoholic fecal samples (whereas it was present in ~80% of the healthy samples), illustrating the alcohol related loss of this analyte. A decrease in isobutyrate was also observed in the home collected alcoholic fecal samples (*p* = 0.006; fold change = 1.72). Overall, the alcohol related decrease of propionate and isobutyrate may be a reflection of the alcohol induced changes to the microbiome composition and could provide a mechanism through which alcohol-induced changes to the microbiota composition contribute to alcohol-induced gut leakiness. However, it is not clear why the other SCFAs remain relatively unaffected.

**Fig 9:**
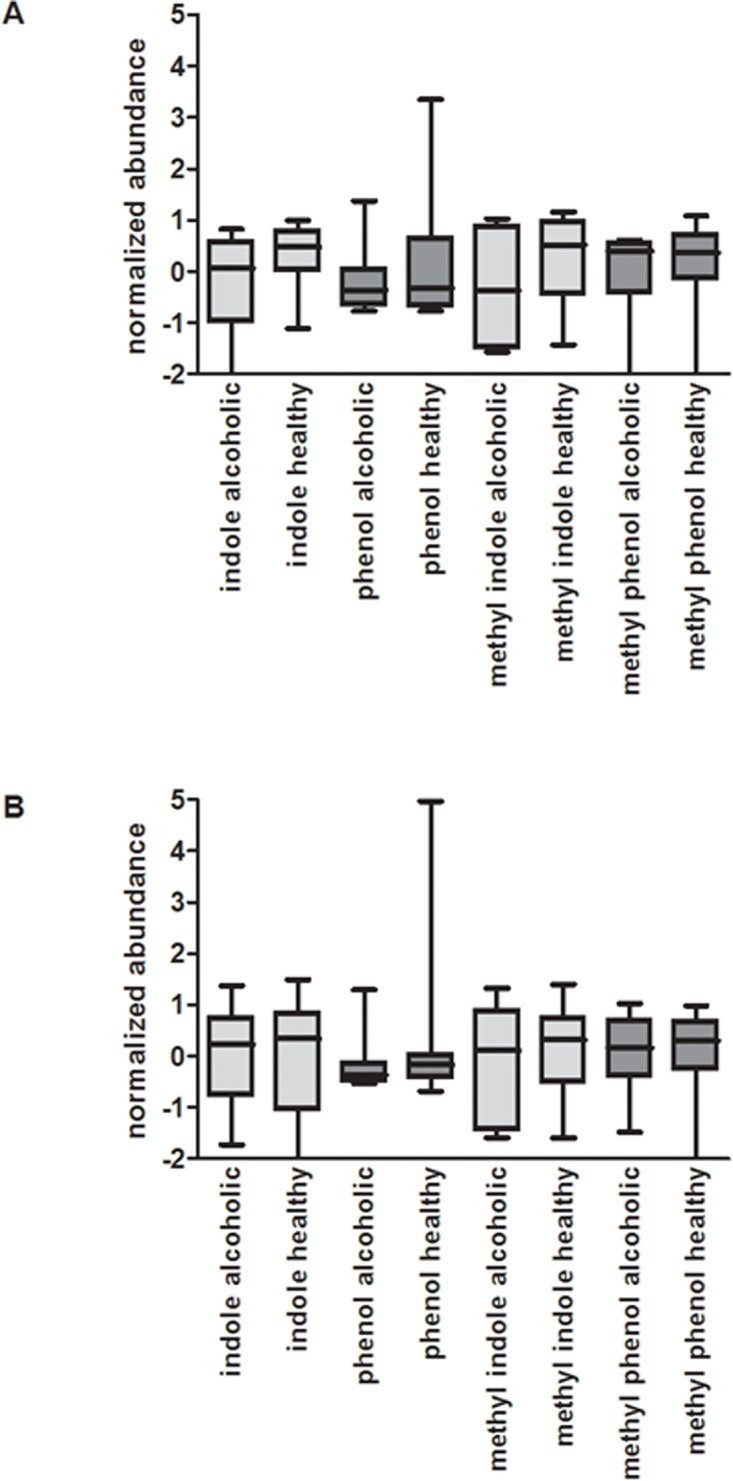
Comparison of protein putrefaction products among the cohorts. Box plots depicting relative metabolite abundance are shown, as described in Fig. 8. A) Among the endoscopy collected samples, with the exception of methyl indole, the metabolites are comparable in abundance within the healthy and alcoholic cohorts. Note however, the difference observed with methyl indole is not statistically significant (*p* = 0.519). B) In the home collected samples, metabolite abundance is very similar between the healthy and alcoholic fecal samples.

In contrast to the SCFAs, protein putrefaction products such as indole, methyl indole, phenol, and methyl phenol have been shown to be injurious to intestinal epithelial cells and could disrupt intestinal barrier integrity [43]. Fig. 9 illustrates box plots depicting the abundance of these metabolites, in both the endoscopy and home collected healthy and alcoholic cohorts. While there is a 1.7 fold decrease in the median abundance of methyl indole in the endoscopy collected alcoholic cohort, relative to the healthy cohort, the difference is not statistically significant (*p* = 0.519). Hence, excessive alcohol consumption appears to have little influence on the abundance of these indicators of protein putrefaction.

As listed in Table 2, nine metabolites were found to be significantly decreased in the home collected alcoholic fecal samples. Of these, caryophyllene, a widely dispersed phytochemical [44–46], is a well characterized agonist of the cannabinoid receptor 2. Interestingly, the association of caryophyllene with the cannabinoid receptor 2 has been shown to reduce voluntary alcohol intake in mice and as such the receptor has been suggested as a target for the pharmaceutical intervention of alcoholism [47]. Within this context, it is noteworthy that the median level of caryophyllene abundance in the alcoholic cohort was found to be twofold lower than that found in the healthy cohort (p = 0.044; see Fig. 7B and Table 2).

The relative abundance of phellandrene and 1-naphthalenol are also decreased in the home collected alcoholic fecal cohort, relative to the healthy group (Table 2 and Fig. 7B). A monoterpenoid, phellandrene is a constituent of many plant extracts [48–50], whereas the naphthalene derivative 1-naphthalenol is implicated as a biomarker for exposure to polycyclic aromatic hydrocarbons [51–53]. While the median abundance of both phellandrene and 1-naphthalenol are significantly decreased in the alcoholic samples relative to healthy (*p* < 0.05, fold change >2), the biological significance and implications of this abundance change remain elusive.

In the home collected fecal samples, the most significant decrease in relative metabolite abundance in the alcoholic cohort is observed with dimethyl disulfide, dimethyl trisulfide, and camphene (all with a fold change >4; Table 2 and Fig. 7B). Microbial products of decomposition [54], dimethyl disulfide and dimethyl trisulfide were detected in all 22 of the tested alcoholic and 24 of the 25 healthy cohort fecal samples. With over a fourfold decrease in median abundance level relative to healthy controls (p < 0.016; Table 2), dimethyl disulfide and dimethyl trisulfide are potential biomarkers of chronic alcohol consumption in the home collected fecal samples. It is noteworthy though, these two analytes do not demonstrate a statistically significant change in the endoscopy collected fecal samples (dimethyl disulfide—*p* = 0.93, fold change = 1.1, dimethyl trisulfide—*p* = 0.28, fold change = 1.3). This may reflect the means by which they are formed (via the oxidation of methanethiol [55,56]), more likely to occur in passaged fecal samples exposed to air. In fact, in the home collected samples, a threefold reduction in median abundance of methanethiol is also apparent in the alcoholic cohort relative to the healthy cohort, whereas in the endoscopy collected samples this fold change is only 1.1. Also of note, there is a 3.5 fold greater median abundance of methanethiol in the home collected healthy samples as there is in the endoscopy collected healthy fecal cohort.

The phytochemical camphene, a bicyclic monoterpene shown to attenuate hepatic steatosis in mice [57], demonstrates a 5.5 fold reduction in median abundance in the alcoholic fecal samples relative to healthy (Table 2 and Fig. 7B). As hepatic steatosis occurs in the early stages of alcohol liver disease, it is interesting to reflect on the significant decrease in camphene levels in the alcoholic samples. There is no statistically significant change in camphene in the endoscopy collected samples.

The metabolite 1-benzoyloxy-2,5-pyrrolidinedione also demonstrates a significant reduction in median abundance in the home collected alcoholic fecal samples (Table 2 and Fig. 7B). Indeed, while 1-benzoyloxy-2,5-pyrrolidinedione was detected in 20 of the 25 healthy fecal samples, the analyte could only be detected in 55% of the alcoholic fecal samples, and with a 2.2 fold reduction in median abundance in the alcoholic cohort (*p* = 0.04). Similarly, (2-aziridinylethyl)amine, detected in 84% of the healthy samples and 73% of the alcoholic samples, is decreased in the alcoholic cohort 2.5 fold relative to the median abundance in the healthy cohort (*p* = 0.03; Table 2 and Fig. 7B). The biological relevance of 1-benzoyloxy-2,5-pyrrolidinedione and (2-aziridinylethyl)amine remains unclear. 6-Methyl-5-hepten-2-one, on the other hand, is a well-known mammalian VOC detected in skin, breath, and fecal samples [58–62]. Identified in 21 of 25 home collected healthy fecal samples, but only 14 of 22 alcoholic samples, 6-methyl-5-hepten-2-one is decreased 3.86 fold in median abundance in the alcoholic cohort (p = 0.033; Table 2 and Fig. 7B). However, 6-methyl-5-hepten-2-one levels have been linked to the estrus cycle in several female mammals [61,63,64]. It’s noteworthy, as indicated in Table 1, the home collected healthy fecal cohort contains samples obtained from 14 female participants, whereas the alcoholic cohort has samples obtained from 4.

### Biomarkers of Alcoholism

As we have demonstrated previously [28], the approach to fecal sample collection has a profound impact on the derived VOC metabolome. Hence, a strong argument can be made in favor of the endoscopy collected samples, as they are immediately snap frozen upon collection and thus best reflect the colonic metabolism occurring *in vivo*. Indeed, in studies seeking to understand the pathology of disease, the value of the endoscopy collected samples is clear. However, our comparative analysis of home collected fecal samples also demonstrates clear differentiation of healthy and alcoholic VOC metabolomes (Fig. 3). Thus, as the home collected samples are drastically cheaper to obtain, they appear well suited to investigations seeking potential biomarkers of disease.

**Fig 10:**
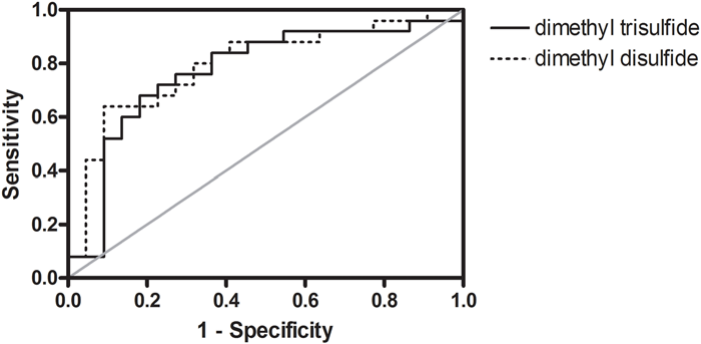
ROC curve of dimethyl disulfide and dimethyl trisulfide. The area under the curve is 0.80 and 0.77, respectively, which are indicative of a fairly good diagnostic test (albeit not excellent).

Ideally, a biomarker of chronic alcohol consumption will appear in all of the healthy and/or alcoholic cohort samples and demonstrate a significant change in abundance between the two cohorts. Accordingly, dimethyl disulfide and dimethyl trisulfide are nearly ubiquitous among all of the home collected fecal samples, yet their relative abundance in the alcoholic feces is significantly suppressed (Table 2). Indeed, their area under the ROC curve is 0.80 and 0.77 respectively, indicative of a marker affording a reasonable balance between sensitivity and specificity (Fig. 10). Additionally, despite its absence in some of the home collected healthy and alcoholic fecal samples, camphene also has an area under the ROC curve indicative of a good biomarker (Table 2). On the other hand, with the endoscopy collected samples, although propanoic acid is ubiquitous among all of the tested samples, the area under the ROC curve is only 0.67, which affords poor accuracy in the determination of alcoholism. Instead, the metabolites 2-tetradecen-1-ol and 6-pentadecen-1-ol are much better biomarkers of alcoholism in the endoscopy collected samples (Table 2). Further studies are now needed to qualify/validate these potential biomarkers.

It is important to consider the impact of diet on the stool VOC profile as dietary products are substrates for bacterial metabolism and thus the type of diet can impact bacterial metabolism. We used a food frequency questionnaire to assess the global dietary habit of our subjects and there was no significant difference between alcoholic and control cohorts in regards to their dietary habit. Thus, the differences in stool VOC profile between alcoholic and control groups cannot be explained by a difference in dietary habit. However, we acknowledge that a recall dietary questionnaire has its limitation and a future longitudinal study with prospective collection of detailed information on diet is required to fully assess the impact of diet on fecal VOCs in alcoholics.

### Summary

In summary, regardless of whether the samples were collected *in vivo* or *ex vivo* after passage, it is clear that excessive alcohol consumption has a significant effect on the composition of the VOC metabolome, as evidenced by the clear cohort separation in the PCA plots, the distinct healthy and alcoholic clades formed with unsupervised hierarchal clustering analysis, and the drastically distinct metabolite correlation networks within the healthy and alcoholic VOC metabolomes. Numerous metabolites undergo a significant fold change in abundance with excessive alcohol consumption, with many found to increase while others decrease in abundance in the alcoholic feces, relative to healthy controls. The most notable metabolite alterations in the alcoholic samples include: (1) an elevation in the oxidative stress biomarker tetradecane; (2) a decrease in five fatty alcohols with anti-oxidant property and a relationship to the abundance of plasmalogens, known to be linked to cell-cell interactions and gap junctions; (3) a decrease in the short chain fatty acids propionate and isobutyrate, important in maintaining intestinal epithelial cell health and barrier integrity; (4) a decrease in alcohol consumption natural suppressant caryophyllene; (5) a decrease in natural product and hepatic steatosis attenuator camphene; and (6) decreased dimethyl disulfide and dimethyl trisulfide, microbial products of decomposition. With this initial insight into alcohol associated VOC metabolomic change, the stage is set for additional studies associating these metabolites with the progression of alcohol associated pathologies and interventional studies directed to correct these abnormalities to determine whether alcohol associated pathologies such as ALD can be prevented.

## Supporting Information

S1 Fig PCA based only on the ten top scoring metabolites, determined by the weight loadings (squared cosines of the variable) in Fig. 3. The resulting three dimensional plot from the endoscopy collected fecal dataset is shown in A) and the home collected fecal dataset is shown in B).
(TIF)

S2 Fig Correlation networks of the endoscopy collected (A) and home collected (B) fecal VOC metabolomes. Pearson’s correlation coefficients were calculated for all metabolites present in at least 21% of the total fecal samples. A Pearson correlation value greater than 0.95 is depicted as a green line between metabolites, while a Pearson correlation value less than-0.95 is depicted as a red line. Metabolites are numerically represented in the network and their placement around the circumference of the network is fixed among the paired plots. Regardless of the approach to fecal collection, the fecal samples from the alcoholic participants have a significantly different correlation network than that seen in the fecal samples from non-alcoholics.
(TIF)
