# Supplementary figures and images for: Alcohol Induced Alterations to the Human Fecal VOC Metabolome
Source: PLoS One. 2015 Mar 9;10(3):e0119362. doi: 10.1371/journal.pone.0119362 (PMC4353727; doi:10.1371/journal.pone.0119362)

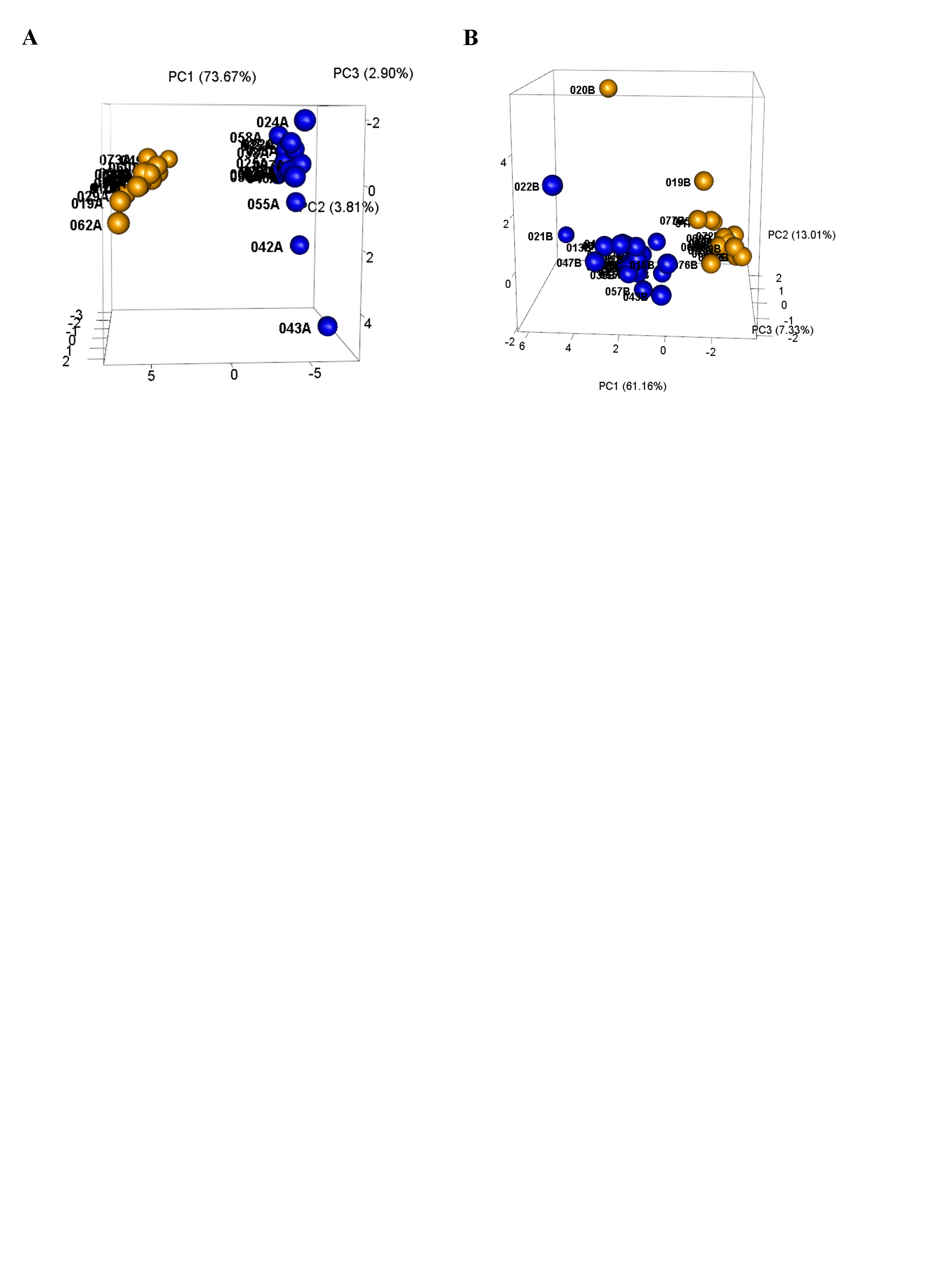

Supplement: S1 Fig — The resulting three dimensional plot from the endoscopy collected fecal dataset is shown in A) and the home collected fecal dataset is shown in B). (TIF) [file pone.0119362.s001.tif]

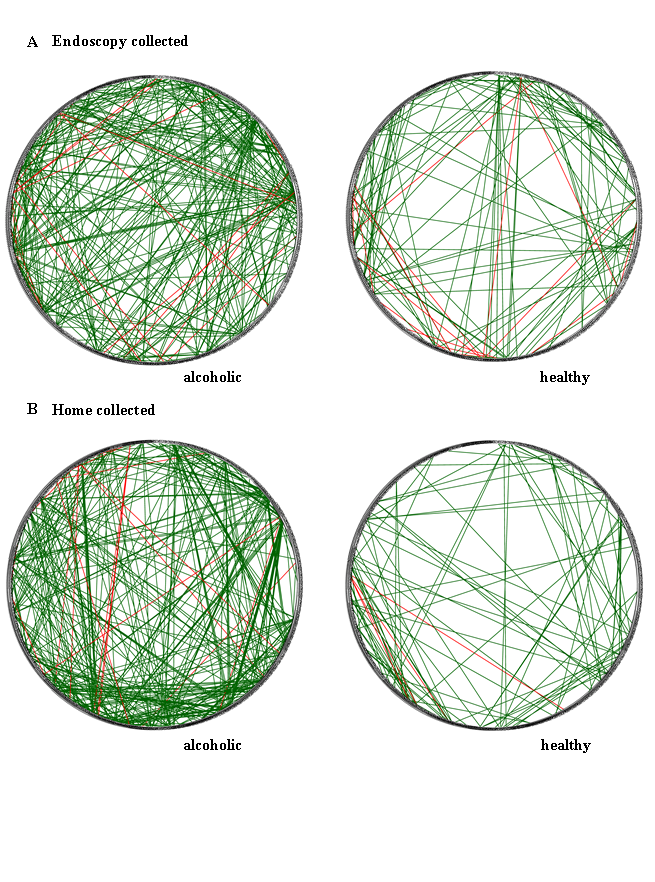

Supplement: S2 Fig — Pearson’s correlation coefficients were calculated for all metabolites present in at least 21% of the total fecal samples. A Pearson correlation value greater than 0.95 is depicted as a green line between metabolites, while a Pearson correlation value less than-0.95 is depicted as a red line. Metabolites are numerically represented in the network and their placement around the circumference of the network is fixed among the paired plots. Regardless of the approach to fecal collection, the fecal samples from the alcoholic participants have a significantly different correlation network than that seen in the fecal samples from non-alcoholics. (TIF) [file pone.0119362.s002.tif]
